# Expression and significance of glucose transporter-1, P-glycoprotein, multidrug resistance-associated protein and glutathione S-transferase-π in laryngeal carcinoma

**DOI:** 10.3892/ol.2014.2752

**Published:** 2014-12-01

**Authors:** ZHONG-PING MAO, LI-JUN ZHAO, SHUI-HONG ZHOU, MENG-QIN LIU, WEI-FENG TAN, HONG-TIAN YAO

**Affiliations:** 1 Department of Otolaryngology, The Second Hospital of Shaoxing City, Shaoxing, Zhejiang 312000, P.R. China; 2 Department of Otolaryngology, The First Affiliated Hospital, College of Medicine, Zhejiang University, Hangzhou, Zhejiang 310003, P.R. China; 3 Department of Pathology, The Second Hospital of Shaoxing City, Shaoxing, Zhejiang 312000, P.R. China; 4 Department of Pathology, The First Affiliated Hospital, College of Medicine, Zhejiang University, Hangzhou, Zhejiang 310003, P.R. China

**Keywords:** laryngeal carcinoma, glucose transporter-1, P-glycoprotein, multidrug resistance-associated protein, glutathione-s-transferase-π

## Abstract

Increasing glucose transporter-1 (GLUT-1) activity is one of the most important ways to increase the cellular influx of glucose. We previously demonstrated that increased GLUT-1 expression was an independent predictor of survival in patients with laryngeal carcinoma. Thus, GLUT-1 may present a novel therapeutic target in laryngeal carcinoma. In this study, the expression of GLUT-1, P-glycoprotein (P-gp), multidrug resistance-associated protein 1 (MRP1) and glutathione S-transferase-π (GST-π) in laryngeal carcinomas was investigated by immunohistochemistry. Additionally, possible correlations between GLUT-1 and P-gp, MRP1 and GST-π and various clinicopathological parameters were analyzed. In this study, 52.9% (18/34), 58.8% (20/34), 20.6% (7/34) and 58.8% (20/34) of the laryngeal carcinomas were positive for GLUT-1, P-gp, MRP1 and GST-π, respectively. The expression of GLUT-1, P-gp, MRP1 and GST-π was higher in laryngeal carcinoma specimens when compared with laryngeal precancerous lesions (P<0.05). Pearson’s correlation analysis showed correlations between GLUT-1 and P-gp (r=0.364; P=0.034), GLUT-1 and MRP1 (r=0.359; P=0.037) and P-gp and GST-π (r=0.426; P=0.012). GLUT-1 expression was found to significantly correlate with tumor-node-metastasis classification (P=0.02) and clinical stage (P=0.037). Furthermore, P-gp was found to significantly correlate with clinical stage (P=0.026). Univariate analysis showed that MRP1 expression was significantly associated with poor survival (c^2^=5.16; P=0.023). Multivariate analysis revealed that lymph node metastasis (P=0.009) and MRP1 overexpression (P=0.023) were significant predictors of poor survival. In the present study, the expression of GLUT-1, P-gp, MRP1 and GST-π in laryngeal carcinomas was investigated, as well as the correlations between these proteins. P-gp was found to significantly correlate with clinical stage, while MRP1 overexpression was significantly associated with poor survival.

## Introduction

The mechanism of carcinoma resistance to chemoradiotherapy may involve multiple factors. Among these, hypoxia is an important factor in the chemoresistance of head and neck carcinomas, such as oral squamous cell carcinoma and nasopharyngeal carcinoma (1,2). Under conditions of hypoxic stress, carcinoma cells require more energy to support cell proliferation. Glucose is an important source of energy. Therefore, increasing glucose transporter-1 (GLUT-1) activity is one of the most important ways to increase the cellular influx of glucose (3). In our previous study, it was demonstrated that increased GLUT-1 expression was an independent predictor of survival in patients with laryngeal carcinoma (4). Thus, GLUT-1 may present a novel therapeutic target in laryngeal carcinoma (5,6). However, few studies have investigated GLUT-1 expression and tumor drug resistance (7–10).

Another important factor in tumor resistance to chemotherapy is intrinsic chemotherapy resistance (11–14). Various drug transporter proteins inside tumor cells are involved in intrinsic chemotherapy resistance, including P-glycoprotein (P-gp), multidrug resistance-associated protein (MRP) and glutathione-s-transferase-π (GST-π). These drug transporters are overexpressed in a number of cancer types, such as liver cancer, lung cancer, glioma and gallbladder cancer (11–14), and overexpression of these proteins is associated with hypoxia (15,16). In the present study, the expression of GLUT-1, P-gp, MRP1 and GST-π in laryngeal carcinomas was investigated by immunohistochemistry (IHC). The present study investigated the correlations between the expression of these proteins, with respect to various clinical and pathological features of laryngeal carcinoma.

## Materials and methods

### Patients and tissues

A total of 34 paraffin-embedded archival tissue blocks from laryngeal squamous cell carcinoma patients were obtained from The Second Hospital of Shaoxing City (Shaoxing, China) between May 2005 and January 2012. A total of 34 paraffin-embedded archival tissue blocks from patients with precancerous lesions were also obtained from The First Affiliated Hospital, College of Medicine, Zhejiang University (Zhejiang, China). A representative paraffin block from each tumor was selected for immunohistochemical analysis. The diagnosis was confirmed after all hematoxylin and eosin-stained sections were reviewed blindly. No patients had received preoperative radiotherapy or chemotherapy. Demographic and clinicopathological data, including gender, age, tumor-node-metastasis (TNM) stage were retrospectively collected. The study protocol was approved by the institutional review board of The Second Hospital of Shaoxing City and The First Affiliated Hospital, College of Medicine, Zhejiang University and all patients provided consent.

### IHC

Formalin-fixed and paraffin-embedded tissue blocks from primary lesions were cut into 4-μm sections, and representative sections were analyzed immunohistochemically using an EliVision™ Plus IHC kit (Fuzhou Maixin Biotechnology Development Co., Ltd., Fuzhou, China) for GLUT-1 (cat. no. ab14683; 1:50) rabbit polyclonal; a mouse monoclonal antibody against P-gp (cat. no. ab3366; 1:100), a mouse monoclonal antibody against MRP1 (cat. no. ab63987; 1:100), and a mouse monoclonal antibody against GST-π (cat. no. ab131059; 1:50; all antibodies purchased from Abcam, Cambridge, MA, USA). Primary antibodies were applied for 1 h at room temperature, and then sections were washed three times with 0.05 mol/l Tris-buffered saline (pH 7.2) and incubated with 50 μl of polymer enhancer (Fuzhou Maixin Biotechnology Development Co., Ltd.) for 20 min. This was followed by incubation with 50 μl polymerized horseradish peroxidase-conjugated anti-mouse immunoglobulin G (Fuzhou Maixin Biotechnology Development Co., Ltd.) for 30 min at room temperature. GLUT-1 expression was considered positive if distinct membrane staining was identified. P-gp, MRP1, and GST-π were identified in the membrane and/or cytoplasm. Protein analysis was performed in 10 random high-power fields; a total of 100 tumor cells were counted from each high-power field for each case and for all antibodies analyzed. The percentage of positive cells was calculated by dividing the number of positive tumor cells by the total number of tumor cells counted. Staining intensity was scored as follows: Negative staining (−), <10% cells were stained positive; weak staining (+), ≥10 but <25% cells were stained positive; moderate staining (++), ≥25 but <75% cells were stained positive; and intense staining (+++), 75–100% cells were stained positive.

### Statistical analysis

Associations between GLUT-1, P-gp, MRP1 and GST-π immunostaining and other parameters were analyzed using the χ^2^ and Fisher’s exact tests. P<0.05 was considered to indicate a statistically significant difference. The associations between GLUT-1 and P-gp, MRP1 and GST-π were analyzed by Spearman’s correlation. Overall survival, which was defined as the time from surgery until mortality from any cause, was plotted as a Kaplan-Meier curve. Univariate survival analysis was performed using the log-rank test and multivariate analysis was performed using Cox proportional-hazards regression analysis. All analyses were conducted using SPSS version 19.0 software (SPSS, Inc., Chicago, IL, USA).

## Results

### Patient characteristics

All patients had squamous cell carcinoma. The subjects included 33 males and one female with a mean age of 62.1 years (range, 45–76 years). A total of 28 (82.4%), five (14.7%), and one (2.9%) patients had tumors located in the glottis, supraglottis and subglottis, respectively. A total of 25 patients received partial laryngetomy (21 vertical partial laryngetomies and four supraglottic partial laryngetomies) and nine patients received total laryngetomy in addition to postoperative radiotherapy. TNM, clinical stage and other clinopathological parameters of the patients are shown in Table I. Six patients were lost to follow-up. Seven patients (20.6%) developed local recurrence and two (5.9%) developed distant metastases. Twenty-two patients were alive at the last follow-up (December 2012). The three- and five-year cumulative survival rates were 76.0 and 61.0%, respectively.

### Expression of GLUT-1, MRP1, P-gp and GST-π

**Figure 1:**
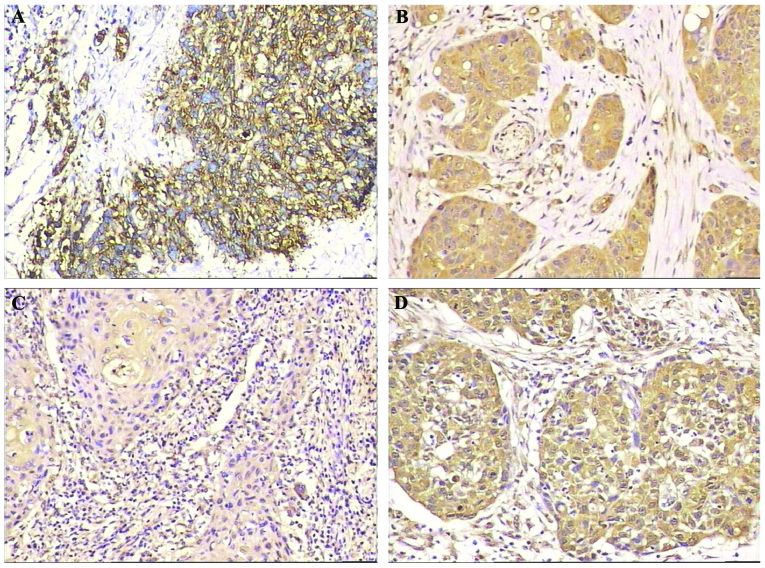
Immunohistochemical analysis of GLUT-1, P-gp, MRP1 and GST-π protein expression in laryngeal carcinoma. Representative positive staining images for (A) Glut-1, (B) P-gp, (C) MRP1 and (D) GST-π. GLUT-1, glucose transporter-1; P-gp, P-glycoprotein; MRP1, multidrug resistance-associated protein 1; GST-π, glutathione S-transferase-π.

In this study, 52.9 (18/34), 58.8 (20/34), 20.6 (7/34) and 58.8% (20/34) of the laryngeal carcinomas were positive for GLUT-1, P-gp, MRP1 and GST-π, respectively (Fig. 1). Pearson’s correlation analysis showed correlations between GLUT-1 and P-gp (r=0.364; P=0.034), GLUT-1 and MRP1 (r=0.359; P=0.037), and P-gp and GST-π (r=0.426; P=0.012).

### Association between GLUT-1, MRP1, P-gp and GST-π expression in laryngeal carcinoma and clinicopathological parameters and prognosis

GLUT-1 expression was found to significantly correlate with TNM stage (P=0.02) and clinical stage (P=0.037). P-gp was found to significantly correlate with clinical stage (P=0.026). No significant difference was identified between GLUT-1 and P-gp expression and the remaining clinicopathological factors investigated. No significant difference was identified between MRP1 and GST-π expression and any of the clinicopathological factors investigated.

**Figure 2:**
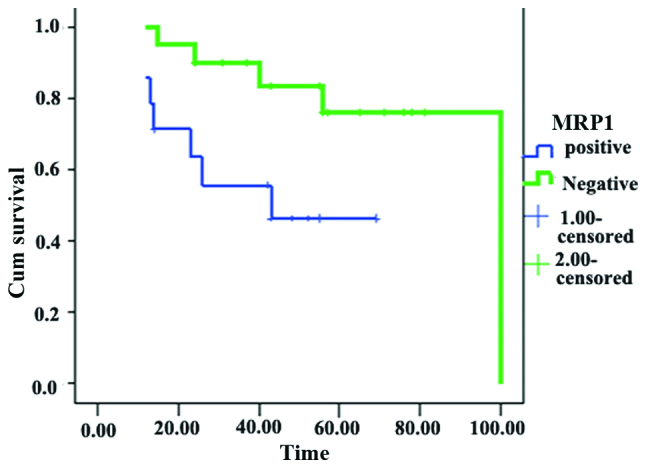
Univariate analysis shows that MRP1-positivity is significantly associated with poorer survival (χ^2^=5.16; P=0.023). MRP1, multidrug resistance-associated protein 1.

Univariate analysis showed that MRP1 expression was significantly associated with reduced survival (χ^2^=5.16; P=0.023; Fig. 2). By contrast, GLUT-1, P-gp and GST-π expression were not associated with survival. Multivariate analysis revealed that lymph node metastasis (P=0.009) and MRP1 overexpression (P=0.023) were significant predictors of poor survival.

## Discussion

P-gp, MRP1 and GST-π are associated with intrinsic chemotherapy resistance (11–14). P-gp and MRP1, two important ATP-binding cassette transporters, affect the intracellular drug concentration by altering drug influx or efflux (12). GST-π is a member of the GST family, which catalyzes the conjugation of glutathione and leads to the inactivation of cytotoxic drugs (12,13). The majority of studies have investigated P-gp, MRPs and GST-π in human solid malignant tumors (11–14). In the present study, P-gp, MRP1 and GST-π expression were investigated in laryngeal carcinomas. The expression of P-gp, MRP1 and GST-π was higher than that in the laryngeal precancerous lesions (P<0.05). Among these proteins, P-gp was found to significantly correlate with clinical stage (P=0.026) and MRP1 overexpression was significantly associated with poor survival (P=0.023). These results are similar to those for other human solid cancers. Yu *et al* (11) found that multidrug resistance protein 3 and MRP1 were poor prognostic factors in liver cancer. In four lung cancer cell lines, SK-MES-1, SPCA-1, NCI-H-460 and NCI-H-446, the expression of P-gp, MRP1 and GST-π was different; the level of GST-π in the SK-MES-1 cells was the highest, whereas the level of P-gp in the SPCA-1 cells was the lowest. The chemoresistance to cisplatin, doxorubicin and VP-16 in the four cell lines was also different; the SPCA-1 cell line was most resistant to cisplatin, and the SK-MES-1 cell line was most resistant to VP-16, but most sensitive to doxorubicin. There was a positive correlation between GST-π expression and resistance to cisplatin, between TopoIIα expression and resistance to VP-16, and a negative correlation was noted between TopoIIα expression and resistance to doxorubicin. Among these proteins, GST-π may be useful for the prediction of intrinsic resistance to cisplatin (12). P-gp and MDR have been found to be highly expressed in gallbladder carcinoma (14). Similarly, P-gp, MRP1 and GST-π were highly expressed in gliomas (13). However, the regulatory mechanism underlying the high level of expression of these proteins in cancer remains unclear.

Previous studies have shown that the overexpression of these proteins may be associated with hypoxia (15,16). Hypoxia is an important factor in chemoresistance (1,2). A small number of studies have demonstrated co-expression of GLUT-1 and P-gp in the capillaries of the blood-brain barrier (18,19). However, the association between GLUT-1, P-gp, MRP1 and GST-π expression in human cancers has not been reported. In the present study, correlations were identified between GLUT-1 and P-gp, GLUT-1 and MRP1 and P-gp and GST-π in laryngeal carcinoma.

In addition, GLUT-1 is associated with a poor response to chemoradiotherapy, and the silencing of GLUT-1 expression may increase sensitivity to chemotherapeutic agents (7–10). Solid cancers grow rapidly and cause hypoxia due to an insufficient supply of blood and oxygen. Under hypoxic conditions, GLUT-1 may supply glucose to meet the energy requirements of cancer cells (4,7–10). In the present study, high levels of GLUT-1 expression were identified in laryngeal carcinomas, which was similar to the results of our previous study regarding laryngeal carcinoma (4). However, GLUT-1 expression was not associated with any clinicopathological parameters. These results differ from our previous and other studies, and these differences may be due to variation in histopathological type, immunohistochemical techniques, tumor stage and sample size (4,10). GLUT-1 expression has been found to be significantly associated with a reduced response to chemoradiotherapy, in oesophageal cancer, rectal cancer and ovarian carcinoma (9). These differences may be due to variation in histopathological type, immunohistochemical techniques, tumor stage and sample size.

In the present study, the expression and correlations between GLUT-1, P-gp, MRP1 and GST-π in laryngeal carcinoma samples was investigated. Whether these proteins are involved in resistance to chemotherapy in patients with laryngeal carcinoma requires further study.

In conclusion, this is the first study to investigate the expression of GLUT-1, P-gp, MRP1 and GST-π in laryngeal carcinomas and the correlations between these proteins. P-gp was found to significantly correlate with clinical stage, while MRP1 overexpression was significantly associated with poor survival. In our future studies, we will further investigate whether these proteins may be resistant to chemotherapy in laryngeal carcinoma *in vivo*. Inhibition of these proteins by targeted treatment may enhance the sensitivity of chemotherapy in laryngeal carcinoma.

## Figures and Tables

**Table I:** Clinicopathological parameters and GLUT-1, P-gp, MRP, and GST-π expression in 34 laryngeal carcinomas.

Pt	Gender	Age (years)	Location	DIF	TNM stage	Treatment	Clinical stage^a^	DM	Follow-up	GLUT-1	MRP1	P-gp	GST-π
1	M	70	Glottis	W-M	T1N0M0	VPL	I	No	12 months, lost	−	−	−	−
2	M	61	Glottis	W	T4N1M0	TL+PR	IV	Yes	24 months, DOD	+	+	+	+
3	M	60	Glottis	W-M	T2N0M0	VPL	II	No	81 months, alive	+	+	−	−
4	M	68	Glottis	W-M	T3N0M0	VPL	III	No	23 months, DOD	+	−	−	+
5	M	55	Subglottis	W	T3N1M0	TL+PR	III	No	78 months, alive	+	+	+	+
6	M	72	Supraglottis	M	T3N0M0	TL+PR	III	No	76 months, alive	+	+	−	+
7	M	76	Glottis	M	T3N1M0	TL+PR	III	No	15 months, DOD	+	+	+	+
8	M	63	Glottis	W	T3N0M0	VPL	III	No	71 months, alive	+	+	+	+
9	M	54	Supraglottis	M	T2N1M0	SL+PR	III	No	69 months, alive	−	−	−	+
10	M	50	Glottis	W-M	T2N0M0	VPL	II	No	43 months, lost	−	−	−	−
11	M	68	Glottis	W	T2N0M0	VPL	II	No	56 months, lost	−	+	−	−
12	M	55	Supraglottis	M	T2N0M0	SL+PR	II	No	65 months, alive	−	+	−	+
13	M	76	Glottis	W	T1N0M0	VPL	I	No	57 months, alive	+	+	−	−
14	M	75	Glottis	W-M	T3N0M0	TL+RP	III	No	57 months, alive	+	+	−	−
15	M	65	Glottis	W	T1N0M0	VPL	I	No	56 months, alive	+	+	−	+
16	M	58	Glottis	W	T2N0M0	VPL	II	No	56 months, alive	−	+	−	+
17	M	72	Glottis	M	T1N0M0	VPL	I	No	55 months, alive	−	+	−	+
18	M	57	Glottis	M	T3N0M0	TL	III	No	52 months, alive	−	−	−	−
19	M	67	Glottis	M	T3N2M0	TL+PR	IV	No	40 months, DOD	+	+	−	+
20	M	69	Glottis	W	T1N0M0	VPL	I	No	55 months, alive	−	−	−	−
21	M	45	Glottis	W	T2N0M0	VPL	II	No	48 months, alive	+	−	−	−
22	M	53	Glottis	W	T4N0M0	TL+PR	IV	No	100 months, lost	+	+	−	−
23	F	54	Glottis	M	T2N0M0	VPL	II	No	43 months, alive	+	+	+	+
24	M	69	Glottis	W-M	T2N0M0	VPL	II	No	42 months, alive	−	−	−	+
25	M	64	Glottis	W-M	T4N0M0	TL+PR	IV	No	42 months, alive	+	−	−	+
26	M	61	Supraglottis	W	T2N0M0	SL+PR	II	No	37 months, alive	−	+	−	+
27	M	48	Supraglottis	W-M	T2N0M0	SL+PR	II	No	26 months, DOD	+	−	−	+
28	M	74	Glottis	W	T2N0M0	VPL	II	No	31 months, alive	−	+	−	−
29	M	65	Glottis	M-P	T1N0M0	VPL	I	No	13 months, DOD	−	+	−	+
30	M	47	Glottis	W	T2N0M0	VPL	II	No	24 months, alive	+	+	+	+
31	M	47	Glottis	W-M	T2N1M0	VPL+PR	III	No	24 months, alive	−	−	−	−
32	M	55	Glottis	W	T2N0M0	VPL	II	No	14 months, alive	−	−	−	−
33	M	71	Glottis	W	T2N0M0	VPL	II	No	14 months, alive	−	−	−	−
34	M	66	Glottis	W-M	T2N2M0	VPL+PR	IV	Yes	12 months, lost	−	−	+	+

a As established by the 7th edition of the International Union Against Cancer TNM classification system, 2007 (17).

M, male; F, female; DIF, differentiation; W, well-differentiated; M, moderately differentiated; TNM, tumor-node-metastasis; −, negative expression; +, positive expression; VPL, vertical partial laryngectomy; TL, total laryngectomy; PR, postoperative radiotherapy; SL; supraglottic laryngectomy; DM, distant metastasis; DOD, died of disease; GLUT-1, glucose transporter-1; MRP1, multidrug resistance-associated protein 1; P-gp, P-glycoprotein; GST-π, glutathione S-transferase-π.
